# Determination of Urinary Creatinine in Washington State Residents via Liquid Chromatography/Tandem Mass Spectrometry

**DOI:** 10.1155/2014/247316

**Published:** 2014-12-31

**Authors:** Caroline E. West, Blaine N. Rhodes

**Affiliations:** The Washington State Department of Health, Public Health Laboratories, 1610 NE 150th Street, Shoreline, WA 98155, USA

## Abstract

A viable, quick, and reliable method for determining urinary creatinine by liquid chromatography/tandem mass spectrometry (LC/MS/MS) was developed and used to evaluate spot urine samples collected for the Washington Environmental Biomonitoring Survey (WEBS): part of the Washington State Department of Health, Public Health Laboratories (PHL). 50 *µ*L of urine was mixed with a 1 : 1 acetonitrile/water solution containing deuterated creatinine as the internal standard and then analyzed by LC/MS/MS. Utilizing electrospray ionization (ESI) in positive mode, the transition ions for creatinine and creatinine-d_3_ were determined to be 114.0 to 44.1 (quantifier), 114.0 to 86.1 (qualifier), and 117.0 to 47.1 (creatinine-d_3_). The retention time for creatinine was 0.85 minutes. The linear calibration range was 20–4000 mg/L, with a limit of detection at 1.77 mg/L and a limit of quantitation at 5.91 mg/L. LC/MS/MS and the colorimetric Jaffé reaction were associated significantly (Pearson *r* = 0.9898 and *R* ^2^ = 0.9797, *ρ* ≤ 0.0001). The LC/MS/MS method developed at the PHL to determine creatinine in the spot urine samples had shorter retention times, and was more sensitive, reliable, reproducible, and safer than other LC/MS/MS or commercial methods such as the Jaffé reaction or modified versions thereof.

## 1. Introduction

Biomonitoring is an important way of evaluating human exposures to selected environmental contaminants and is becoming more widely used in public health work [1]. Urine is a widely used matrix in biomonitoring and other clinical testing. Creatinine is often used for normalizing or adjusting urinary analyte concentrations for dilution in clinical samples and is considered an integral part of monitoring for exposures. Creatinine is also commonly used to normalize concentrations of absorbed chemicals in spot urine collection studies like the survey conducted by WEBS [2–4]. The reference ranges for creatinine can vary from source to source. The guidelines of the World Health Organization (WHO) set the occupational range at 30 to 300 mg/dL and the US Department of Transportation measures urinary creatinine down to 5 mg/dL to correct for selective drugs of abuse. There are studies that point out that the limit may need to be lowered to include current environmental toxicants which are now being measured at very trace levels [3, 4]. We set our lower calibration range at 20 mg/L (2 mg/dL).

There are several published analytical methods for measuring creatinine in urine such as the Jaffé reaction [5–7], High Performance Liquid Chromatography (HPLC) [8–10], enzymatic methods [11, 12], and Liquid Chromatography Tandem Mass Spectrometry (LC/MS/MS) techniques [13–15]. Some of the advantages using the LC/MS/MS method developed at the PHL compared to other LC/MS/MS methods include improved linearity, within-day and between-day precision, and lower injection volumes (less system stress). Measuring creatinine by LC/MS/MS technology is much more sensitive and selective. Enzymatic methods and colorimetric methods such as the Jaffé reaction are less specific and can be affected by several interfering substances giving results that are too high. For example, erroneously high values can result in these methods from the conditions of the hydrolysis step where urea and sugar combine to form products which also react with picric acid in the Jaffé reaction. The LC/MS/MS method is not adversely affected by urea, glucose concentrations, or other endogenous substances as discussed elsewhere [5–7].

Many of the methods utilized by the Center for Disease Control and Prevention (CDC) for measuring environmental analytes for biomonitoring are being developed and analyzed with LC/MS/MS technology. Being able to also use LC/MS/MS method for measuring creatinine would eliminate the need to purchase specialized equipment for creatinine analysis.

This study was designed to develop a robust, specific, and sensitive method for analyzing creatinine by LC/MS/MS. It was applied to spot urine samples collected by WEBS and used to normalize the results for pesticide, metal, and other environmental toxicant exposures. A method comparison study performed with the University of Washington (UW) Medical Center's Medicine Reference Laboratory Services (which uses a Beckman coulter Unicel DxC 800 system that runs an automated Jaffé reaction) shows a strong positive correlation between the colorimetric Jaffé reaction and the LC/MS/MS method.

## 2. Materials and Methods

### 2.1. Chemicals and Urine Sample Preparation

All chemicals were of analytical or HPLC grade from Fisher Scientific (Fairlawn, NJ). Creatinine (SRM 914a) was from Standard Reference Materials Program National Institute of Standards and Technology (NIST, Gaithersburg, MD) and creatinine-d_3_ was from Fisher Scientific (Fairlawn, NJ). Water (deionized ≥ 18 MΩ) was purified by a NANOpure Infinity Ultrapure water system (Barnstead, Dubuque, IA) and the carrier grade nitrogen gas was supplied by a Peak Scientific Lab Gas Generator (Billerica, MA). A total of 1576 WEBS spot urine samples were collected during two different exposure studies and stored at ≤−70°C until analysis. 626 samples were collected to assess for the exposure of pyrethroid metabolites in pesticide applicators. 950 samples were collected to assess for the exposure to pyrethroid metabolites, bisphenol A (BPA), and phthalate metabolites in low income households. Both studies were reviewed and approved by the Washington State Institutional Review Board. Frozen samples were thawed at room temperature and sonicated for 10 minutes. After sonication, the urine was vortexed for 20 seconds. 50 *μ*L of urine was spiked with 450 *μ*L of the creatinine-d_3_ internal standard solution (ISTD), mixed again and analyzed via LC/MS/MS. The ISTD was prepared by weighing out 0.0155 g ± 0.005 g of the creatinine-d_3_ powder, transferring it to a 500 mL volumetric flask and filling it to volume with a solution of 1 : 1 acetonitrile/18 MΩ DI water.

### 2.2. Tandem Mass Spectrometry

The LC/MS/MS analysis was performed on an Agilent 1200 HPLC stack coupled to an Agilent 6410A triple quadrupole mass spectrometer equipped with an electrospray ionization source (ESI). The HPLC included an in-line degasser, binary pump, temperature controlled column compartment, and two 54-vial tray autosampler racks. The analysis was performed in positive ion mode with a +4000 V charge on the capillary. The gas temperature was set to 100°C with a flow of 12 L/min. The nebulizer was set to 40 psi and the electron multiplier voltage (EMV) was set at 0. The acquisition method utilized multiple reaction monitoring scanning (MRM) with a dwell time of 200 ms, a Fragmentor Voltage of 110 V, and Collision Energies at 20 V for the Creatinine Quantifier and Internal Standard transitions and 8 V for the Qualifier transition. Ultra high purity nitrogen gas was used as the collision gas. A MacMod ACE 3 C-18 column, 3.0 × 100 mm, 3.00 *μ*m (column: part number ACE-111-1003), was used with a flow rate of 0.5 mL/min at ambient temperature. Isocratic separation was achieved using 75% acetonitrile containing 0.1% formic acid and 25% 18 MΩ water containing 0.1% formic acid. 1 *μ*L of the sample (or standard) containing the internal standard was injected onto the column.

The retention time was 0.85 minutes and was set to run at 4 minutes per sample. Mass Hunter Quantitative software was used for peak integration and data analysis. Results from samples were calculated off of the calibration curve which was constructed from the peak area ratios of the analyte to the internal standard for each level.

### 2.3. Linearity

The linearity of this method was determined by preparing eight aqueous calibration standards and analyzing nine of our archived College of American Pathology (CAP) proficiency testing (PT) urine samples in ten separate runs and then calculating percent recoveries based on the CAP assigned mean values for each sample. The calibrators were prepared by serially diluting them with 18 MΩ DI water at the following concentrations: 20, 50, 200, 500, 1000, 2000, 3000, and 4000 mg/L. The calibration curve was constructed using the ratio of the peak area of the creatinine to the peak area of the ISTD plotted against the calibration concentrations with a 1/*x* weighting applied.

### 2.4. Between-Day Precision and Accuracy

The between-day precision was measured and the percent relative standard deviation (RSD) was established at 3 quality control (QC) levels over several days during validation. Accuracy was established at the same time using the mean of each QC level and applying ±3*σ*.

### 2.5. Within-Day Precision and Interferences/Recovery

A standard addition experiment was designed to establish the within-day precision for the method as well as monitor percent recoveries looking for interferences or recovery issues. 50 *μ*L of the 3000 mg/L solution was added to 50 *μ*L of the QCL urine and mixed well to create a spiked QC solution. 50 *μ*L of the spiked QC solution was transferred into an autosampler vial and 450 *μ*L of ISTD solution was added. The sample was then mixed well and 10 replicate injections of the spiked QC were analyzed.

### 2.6. Detection Limits and Reproducibility

The Limit of Detection (LOD) and Limit of Quantitation (LOQ) were calculated from the concentrations of the lowest calibrator (20 mg/L) for each of the 20 validation runs.

Validation was established based on CDC modified Westgard Rules. The LOD was calculated statistically as 3 × the standard deviation and the LOQ at 10 × the standard deviation. The reproducibility was established by calculating the average results for each QC level established by 2 different analysts during validation. The average of the first analyst was subtracted from the average of the second analyst, divided by the target concentration, and multiplied by 100 to establish the percent error.

### 2.7. Method Comparison Study

A comparison study with the UW Medical Center's Medicine Reference Laboratory Services was conducted to evaluate the reliability of the LC/MS/MS method. 50 participant urine samples were homogenized and 2 aliquots per sample were separated for this study. A set of samples was analyzed in-house using the LC/MS/MS method and the duplicates were analyzed at the UW Medical Center using an automated Jaffé reaction. The percent recoveries were calculated based on the UW values as the target values and a Bland-Altman plot was constructed using the results and consisted of the averages of the differences and the 95% limits of agreement for the 2 methods.

### 2.8. Statistical Analysis

The Bland-Altman plot was constructed using Excel 2007 software. Correlations between the LC/MS/MS and the Jaffé reaction methods were established using Least Squares Regression analysis in Excel 2007. Analyses were also conducted in R (R Development Core Team, 2013) to verify the correlation results. Both statistical programs were in agreement.

### 2.9. Results and Discussion

**Figure 1:**
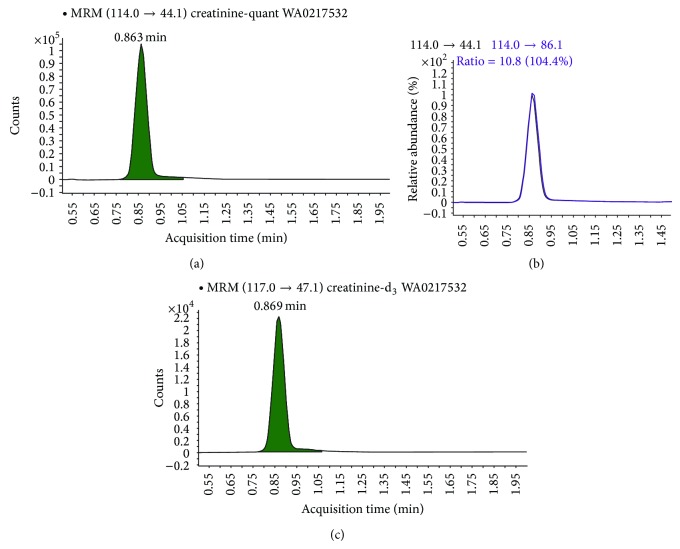
Chromatogram of a WEBS participant urine sample showing the transition ions for creatinine and creatinine-d_3_: 114.0 to 44.1 (quantifier; (a)), 114.0 to 86.1 (qualifier; (b)), and 117.0 to 47.1 (creatinine-d_3_; (c)).

The linear calibration range was 20–4000 mg/L. The transition ions for creatinine and creatinine-d_3_ were determined to be 114.0 to 44.1 (quantifier), 114.0 to 86.1 (qualifier), and 117.0 to 47.1 (creatinine-d_3_) under the ESI ionization conditions as shown in Figure 1. The retention time for creatinine was 0.85 minutes and the total run time per sample was set to 4 minutes.

Three WEBS participant urine samples (Table 1), including the one shown in Figure 1, were chosen to demonstrate the difference between creatinine corrected and noncorrected results for selected pyrethroid metabolite pesticides, 3,5,6-trichloro-2-pyridinol (an Organophosphate insecticide), selected phthalate pesticide metabolites, and bisphenol A.

**Figure 2:**
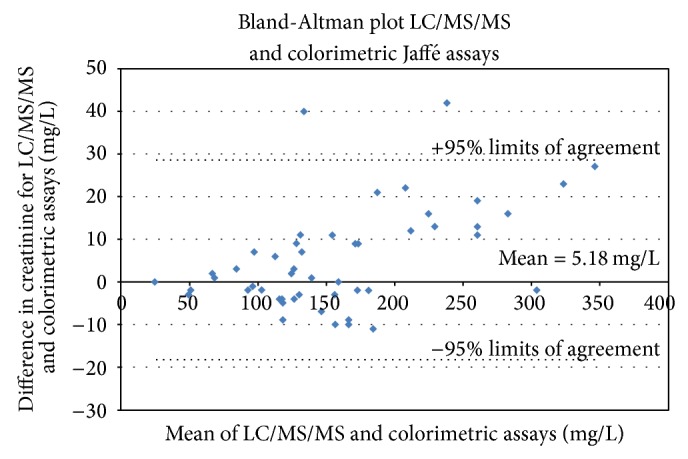
Bland-Altman plot comparing the LC/MS/MS and Jaffé reaction methods for 50 urine samples.

The Bland-Altman analysis showed that there was no systematic difference between the LC/MS/MS and the colorimetric Jaffé reaction and only 4% of the points (2 out of 50) were outside of the 95% limit of agreements calculated as the mean of the difference ± 1.96 times the standard deviation as shown in Figure 2.

### 2.10. Linearity

The average coefficient of correlation (*R* ^2^) for this method was 0.9999. The signal to noise (*S*/*N*) ratio was at least 10 : 1 and the qualifier ratios were within ±20% of the target ratio.

Linearity of the standard curve extended over the entire calibration range. The averages of the percent recovery values were all between 80 and 120% which showed excellent linearity. The results for linearity for the PHL method compared to other LC/MS/MS methods researched for this paper was compiled in Table 2.

### 2.11. Between-Day Precision and Accuracy

The precision was established by calculating the percent RSD for three quality control (QC) levels in urine over the 20 validation runs. The QCL was 1.34%, the QCM was 1.64%, and the QCH was 2.59%. The accuracy ranges for QCL, QCM, and QCH were 580.00–628.71, 775.59–855.67, and 1039.48–1214.74 mg/L, respectively. A comparison of between-day precision values for the PHL method compared to other LC/MS/MS methods referenced in this paper was compiled in Table 3.

### 2.12. Within-Day Precision and Interferences/Recovery

The within-day precision was established during the standard addition experiment. The target concentration was 1802.18 mg/L of creatinine. The average of the 10 replicates was 1861.72 mg/L with a standard deviation of 6.17, a percent RSD of 0.33%, and a percent recovery of 103.30%. The comparisons for the within-day precision values are shown in Table 4.

### 2.13. Limits of Detection and Reproducibility

The LOD was 1.77 mg/L and the LOQ was 5.91 mg/L. The reproducibility was calculated for the QCL, QCM, and QCH during validation. The percent errors were 0.53%, 0.22%, and 3.78%, respectively.

### 2.14. Method Comparison Study

**Figure 3:**
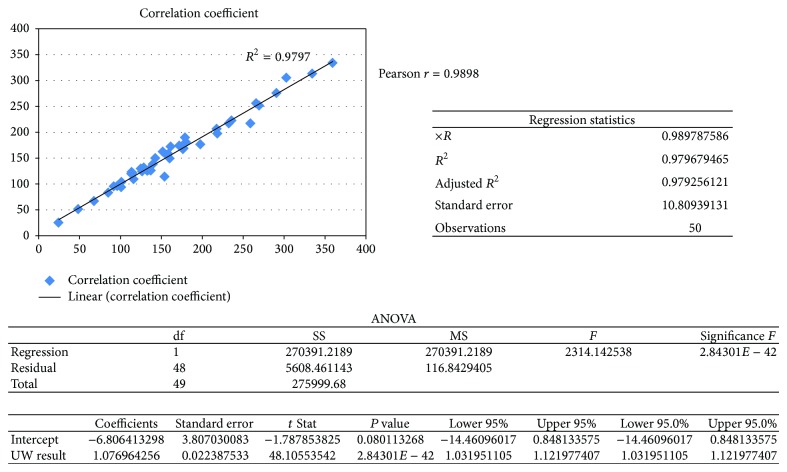
Correlation between LC/MS/MS in-house analysis of urine and UW Medical Center's analysis of duplicate urine samples using an automated Jaffé reaction.

49 of the 50 samples were within ±20% of the theoretical value showing excellent comparability. The correlation between the 2 methods showed the LC/MS/MS and the colorimetric Jaffé reaction were associated significantly (Pearson *r* = 0.9898 and *R* ^2^ = 0.9797, *ρ* ≤ 0.0001) as shown in Figure 3.

## 3. Conclusion

The purpose of the study was to develop an in-house method for measuring urinary creatinine in random spot urine samples using preexisting LC/MS/MS instrumentation to correct environmental contaminant concentrations affected by the hydration levels of the participating individuals. This study has shown that the PHL method is selective, robust, and accurate. It has shown improved linearity, within-day precision, and between-day precision values compared to other published LC/MS/MS methods. One limitation, however, is that, with the simplicity of the sample preparation (no cleanup steps), the instrument tends to get dirtier quicker and may require more cleaning. The 1 *μ*L injection volume helps relieve some of that stress to the system. The PHL method not only eliminates the need to use hazardous chemicals like picric acid; it also diminishes affects from endogenous interferences that plague these other techniques. In summary, the LC/MS/MS method developed for the analysis of urinary creatinine in this study has been shown to be quick, simple, and reliable for use in normalizing spot urine samples from exposure biomonitoring surveys.

## Figures and Tables

**Table 1:** Selected WEBS urine samples showing creatinine corrected results and noncorrected results for selected pyrethroid pesticide metabolites: 3-PBA (3-phenoxybenzoic acid) and trans-DCCA (trans-3-(2,2-dichlorovinyl)-2,2-dimethylcyclopropane-1-carboxylic acid); TCPy (3,5,6-trichloro-2-pyridinol); selected phthalate metabolites: MEP (mono-ethyl phthalate), MBP (mono-n-butyl phthalate), MBzP (mono-benzyl phthalate), and MEHP (mono-2-ethylhexyl phthalate); and BPA (bisphenol A).

WEBS participant urine sample	WA0217528	WA0217530	WA0217532
Creatinine (mg/L)	2288.65	2868.16	1408.12
3-PBA (*μ*g/L)	0.56	1.35	0.27
3-PBA (*μ*g/L) creatinine corrected	0.25	0.47	0.19
trans-DCCA (*μ*g/L)	0.34	1.76	0.17
trans-DCCA (*μ*g/L) creatinine corrected	0.15	0.62	0.12
TCPy (*μ*g/L)	1.10	1.78	1.55
TCPy (*μ*g/L) creatinine corrected	0.48	0.62	1.10
MEP (*μ*g/L)	419.00	611.20	21.01
MEP (*μ*g/L) creatinine corrected	183.08	213.10	14.92
MBP (*μ*g/L)	8.83	16.91	9.08
MBP (*μ*g/L) creatinine corrected	3.86	5.90	6.45
MBzP (*μ*g/L)	19.41	23.33	19.56
MBzP (*μ*g/L) creatinine corrected	8.48	8.13	13.89
MEHP (*μ*g/L)	11.64	10.47	10.01
MEHP (*μ*g/L) creatinine corrected	5.08	3.65	7.11
BPA (*μ*g/L)	0.00	2.83	0.00
BPA (*μ*g/L) creatinine corrected	0.00	0.99	0.00

**Table 2:** Comparison of linearity for the Washington State Department of Health (PHL) method and three LC/MS/MS methods referenced in this paper.

Article referenced	Linearity (*R* ^2^)
PHL method	**0.9999**
Reference [13]	**0.9992**
Reference [14]	**0.9995**
Reference [15]	**0.9995**

**Table 3:** Comparison of between-day precision values for the Washington State Department of Health (PHL) method and three LC/MS/MS methods referenced in this paper.

Article referenced	Between-day precision (% RSD)
PHL method	**1.34 to 2.59**
Reference [13]	**2.0 to 4.4**
Reference [14]	**<6**
Reference [15]	**1.5 to 2.9**

**Table 4:** Comparison of within-day precision values for the Washington State Department of Health (PHL) method and three LC/MS/MS methods referenced in this paper.

Article referenced	Within-day precision (% RSD)
PHL method	**0.33**
Reference [13]	**1.1 to 4**
Reference [14]	**0.86 to 8.86**
Reference [15]	**1.0 to 1.8**
